# Reframing Antimicrobial Stewardship as Systems Governance: Aligning Regulation, Diagnostics, Antibiotic Use Measurement, and Surveillance to Address Antimicrobial Resistance

**DOI:** 10.3390/antibiotics15090871

**Published:** 2026-09-07

**Authors:** Bindu Mayi, Dhanya Dharmapalan, Anita Shet

**Affiliations:** 1 Illinois College of Osteopathic Medicine, The Chicago School, Chicago, IL 60607, USA; 2 Pediatric Infectious Diseases, Apollo Hospitals, Navi Mumbai 400614, Maharashtra, India; 3 Department of International Health, Johns Hopkins Bloomberg School of Public Health, Baltimore, MD 21205, USA

**Keywords:** antimicrobial stewardship, antimicrobial resistance, systems governance, diagnostic stewardship, antibiotic use measurement, resistance surveillance, one health, infection prevention and control, health systems

## Abstract

Antimicrobial stewardship (AMS) is often framed as a set of prescribing-focused interventions aimed at optimizing antibiotic selection, dose, route of administration, and duration. In practice, AMS also functions as a systems discipline. Embedded within governance and regulatory frameworks, diagnostic capacity, antimicrobial consumption and use patterns, and surveillance infrastructure, AMS operates within real-world structural and economic constraints, such as resource limitations, conflicting pharmaceutical market incentives, and health system fragmentation. In lower- and middle-income countries, variation in regulatory enforcement, prescribing patterns, diagnostic limitations, and surveillance infrastructure can undermine otherwise well-designed stewardship policies. This paper frames AMS as a systems governance function and proposes the Aligned AMS Systems Governance framework, comprising four core AMS functions—governance and regulation, antimicrobial consumption and antimicrobial use measurement, diagnostic stewardship, and antimicrobial resistance (AMR) surveillance and reporting—within four broader enabling domains: infection prevention and control, public engagement and awareness, environmental stewardship, and One Health coordination. This paper is an analytical narrative review of peer-reviewed literature, global policy frameworks, and implementation guidance. Using India as an illustrative example, we show that inconsistent regulatory enforcement, non-prescription antibiotic access, diagnostic constraints, and pharmaceutical market dynamics interact to complicate stewardship implementation despite the presence of national policies. Effective AMS requires deliberate alignment of these multiple functions and domains through defined responsibilities, routine data collection and sharing across AMS functions, and continuous feedback loops linking findings to coordinated action and subsequent reassessment. Reframing AMS as systems governance clarifies actionable leverage points for implementation and supports more practical, scalable approaches to mitigating AMR across diverse health system contexts.

## 1. Introduction

Antimicrobial stewardship (AMS) is often framed as a set of coordinated, prescriber-focused interventions that optimize antibiotic selection, dosing, route of administration, and duration to improve patient outcomes and limit antimicrobial resistance (AMR) [1]. In practice, AMS is not solely a prescribing problem but is also a systems alignment challenge [2]. Across health systems, antimicrobial consumption is shaped by multiple complex components such as regulatory structures, healthcare access patterns, diagnostic infrastructure, and surveillance systems, together with the feedback loops that connect them. When not intentionally aligned, these components function mostly in isolation, resulting in fragmented AMS efforts. AMR is shaped by multiple system-level factors across health systems and more broadly across human, animal, and environmental sectors. It is therefore a One Health challenge requiring coordinated stewardship across systems and levels of scale, from local to global [3]. Understanding AMS and AMR as systems problems requires examining how regulation, diagnostic capacity, antimicrobial use, surveillance, and other system components influence one another rather than treating antimicrobial use as a series of isolated decisions.

Studies and health-systems analyses have applied systems thinking to AMS by (i) emphasizing integration across system components; (ii) identifying unintended or non-linear effects of interventions such as those seen when restriction policies shift prescribing to other antimicrobial classes or when expanded diagnostic capacity initially increases antimicrobial use (AMU) before improving use of targeted ones; and (iii) demonstrating the importance of feedback loops in revising prescribing guidance and responding to resistance patterns [4,5,6]. The systems thinking framework positions AMU not as an isolated clinical decision but as an emergent property of interconnected processes that span clinical, organizational, and policy domains. Our paper frames AMS as a coordinated systems function that emphasizes alignment of multiple strategies such as regulatory structures, diagnostic capacity, AMU measurement, and resistance surveillance, to improve AMU and reduce the risk of emergence and spread of AMR. Existing international frameworks similarly recognize that effective AMR responses require coordinated action across multiple health-system functions. The WHO Global Action Plan on AMR links optimization of AMU with surveillance, infection prevention and control (IPC), awareness and education, and sustainable investment [2]. More recently, the WHO people-centred approach to addressing AMR in human health brings together governance and accountability, AMR and antimicrobial consumption (AMC)/AMU surveillance, IPC, diagnostic stewardship, AMS, regulation of non-prescription antimicrobial sales, and other interventions within a broader health-system response to AMR [7]. The people-centred approach explicitly recognizes interdependencies among these interventions [7]. At present, coordination among regulatory oversight, diagnostic capacity, AMU measurement, and resistance surveillance remains uneven globally, with particularly pronounced challenges in many lower- and middle-income countries (LMICs) [8,9,10,11,12,13,14]. A systems framing must therefore include specifying how responsibilities are assigned, data are collected and shared, findings are reviewed, and resulting decisions are implemented. Existing frameworks identify many of the components required for effective AMS and AMR control, but generally provide less detail on how these functions can be operationally connected through shared indicators, routine information exchange, multidisciplinary review, documented action, and subsequent reassessment.

We therefore propose the Aligned AMS Systems Governance framework, which organizes AMS around four core functions—governance and regulation, AMC and AMU measurement, diagnostic stewardship, and AMR surveillance and reporting—and four broader enabling domains: IPC, public engagement and awareness, environmental stewardship, and One Health coordination. Within this framework, systems governance refers to how responsibility for these functions is assigned and how the information they generate is shared and used to inform coordinated decisions and action, with subsequent reassessment providing feedback to the system. The framework is intended to complement existing systems-oriented approaches by focusing specifically on how these AMS functions can be connected in practice rather than implemented as separate activities.

## 2. Results

### 2.1. Why Antimicrobial Stewardship Is a Systems Governance Problem

In many LMIC settings, including India, antibiotics classified as “prescription-only” under national drug regulations continue to remain accessible without clinical evaluations, typically through community pharmacies driven by patient demand, time and cost barriers, and uneven enforcement of regulatory schedules [15,16,17]. Such access patterns frequently lead to empiric antibiotic use for viral upper respiratory tract infections and other self-limited conditions, increasing unnecessary exposure to broad-spectrum agents and contributing to avoidable selection pressure on AMR [18,19]. Prior antibiotic exposure is a well-established risk factor for potentially life-threatening *Clostridioides difficile* infection (CDI), with community-onset CDI also documented in Indian surveillance cohorts [20,21]. Community antimicrobial use may also have implications for hospital-onset CDI. In a population-based U.S. study, community antimicrobial claims were associated with hospital-onset CDI rates, supporting further evaluation of stewardship approaches that extend beyond hospital settings [22]. Therefore, stewardship cannot be confined to tertiary hospitals but must also be operationalized across the additional points of antibiotic access and clinical decision-making, including community pharmacies and primary care settings, and through public engagement practices.

Operationally, this requires embedding stewardship into existing workflows rather than adding it as a separate programme. At a systems level, this integration can be anchored within National Action Plans on AMR and facility-level governance structures, where AMS, Infection Prevention and Control (IPC), pharmacy, and clinical leadership share responsibility for implementation and oversight [8,9,10].

In healthcare facilities, governance mechanisms link stewardship teams with IPC committees, using shared indicators such as healthcare-associated infection rates, hand-hygiene compliance, blood culture contamination rates, and antibiotic consumption by ward or syndrome to review whether rising infection burden is driving preventable antibiotic exposure [8,9]. In community and primary care settings, public engagement and awareness practices can be strengthened when they are integrated as simple, repeatable measures into routine encounters, including standardized counselling within clinics and pharmacies on when antibiotics are not needed and why broader-spectrum agents should not be requested [8,10]. As part of environmental stewardship, unused antibiotics should also be returned or discarded safely rather than stored for self-medication or thrown away with general waste [23].

A small set of indicators that can be collected through existing records and workflows should be tracked over time. Depending on the setting, clinic staff, pharmacists, laboratory personnel, IPC staff, or designated stewardship personnel could document measures such as patient counselling at clinic and pharmacy encounters; rates of non-prescription antibiotic requests and dispensing in community pharmacy settings; volumes of antibiotic return or disposal through pharmacy or facility-based programmes; and trends in antibiotic dispensing for self-limited syndromes [9]. The responsibility for collection and review of these indicators should be clearly assigned within the facility, pharmacy network, district health authority, or other organization overseeing the programme. These structured reviews should be used to identify strengths as well as gaps, refine workflows, and target bottlenecks in AMS activities such as incomplete documentation, delayed diagnostic reporting, Access antibiotic stock-outs, continued non-prescription dispensing, or lack of follow-up after counselling [9,24]. These reviews can guide targeted changes intended to reduce unnecessary antibiotic exposure, address preventable infection risks, and improve appropriate use of first-line Access antibiotics.

### 2.2. Regulatory Architecture, Access, and Market Fragmentation

India’s Drugs and Cosmetics Act and Rules provide a formal regulatory architecture for controlled use of medicines, including H, H1 and X classifications that restrict the sale of specified drugs without prescription or additional documentation requirements [16,25]. Schedule H includes prescription-only medicines; Schedule H1 applies additional record-keeping and dispensing requirements to a subset of medicines, including specified antibiotics; and Schedule X applies more stringent controls to specified drugs, including additional prescription, licensing, storage, and record-keeping requirements [26]. However, the existence of a regulatory structure does not guarantee consistent implementation. Enforcement in India is variable across state and central authorities; market incentives may favour higher-margin antibiotics, and antibiotic fixed-dose combinations (FDCs), including some combinations of multiple antibiotics, remain widely used, illustrating how governance gaps can translate into uneven stewardship [16,25,27]. Under India’s national regulatory structure, drug-control functions are split across central and state government authorities. The Central Drugs Standard Control Organization sets national regulatory standards and provides central approval for new drugs, while state drug-control authorities issue licenses and conduct much of the inspection and enforcement activity across distribution and retail systems [28,29,30]. This division complicates AMS because although the central government establishes and mandates the prescription-only rules, day-to-day enforcement of these rules depends on state-level inspection capacity, local priorities, and the feasibility of monitoring a large, fragmented pharmacy market [31,32]. India has an estimated 800,000 pharmacies and drug shops, with less than 4% operating as chains, making routine inspection and regulatory oversight resource-intensive and leaving many outlets exposed to profit incentives that may favour higher-margin antibiotic sales [33,34].

Regulatory architecture alone is insufficient unless it is supported by consistent monitoring, defined responsibility for enforcement, and pharmaceutical market incentives that favor the availability and appropriate use of evidence-based antibiotics over unnecessary broad-spectrum agents and irrational FDCs. In India, enforcement of prescription-only sale requirements and Schedule H1 record-keeping obligations is complicated by persistent non-prescription antibiotic sales in community pharmacy settings [16,25]. India has been reported to have one of the highest levels of market availability of antimicrobial FDCs globally, many of which have been marketed without approval from the Central Drugs Standard Control Organization and have remained on the market despite being unapproved, banned, or lacking sufficient evidence of rationality [27,32,35,36].

Within the proposed framework, regulatory architecture is linked to stewardship measurement and feedback through targeted inspection of high-volume pharmacies; routine review of Schedule H1 compliance where feasible; monitoring sales patterns of irrational or non-recommended FDC antibiotics as well as Watch antibiotics, which have higher resistance potential and are therefore prioritized by WHO for AMS oversight [37]; and use of these data by multidisciplinary AMS teams to continually refine regulatory, pharmacy, clinical, public health, and public-engagement interventions. This connects pharmaceutical oversight to the broader AMS system rather than treating regulatory enforcement as a stand-alone legal function.

### 2.3. AMC and AMU Measurement as a Stewardship Function

Globally, the 2015 World Health Assembly endorsement of the WHO Global Action Plan (GAP) on antimicrobial resistance elevated AMR from a clinical issue to a coordinated policy priority [2]. The GAP’s objectives of awareness, surveillance, IPC, optimization of antimicrobial use and sustainable investment provide the macro-level architecture for national stewardship efforts. However, transitioning from policy to practice requires operational tools. The AWaRe classification, introduced by WHO in 2017, is one such strategic mechanism, providing a measurable stewardship framework by categorizing antibiotics into Access, Watch and Reserve groups to optimize antibiotic use and curb resistance [37]. As organized in the AWaRe framework, Access antibiotics are recommended as first- or second-choice options for many common infections and are considered to have lower resistance potential than Watch and Reserve agents [37,38]. Countries were initially encouraged to aim for ≥60% of national antibiotic consumption of Access agents, with recent recommendations supporting a ≥70% benchmark to curb the growing overuse of broad-spectrum antibiotics [37,39]. AWaRe classification can be applied to AMC and AMU data to measure the relative use of Access, Watch, and Reserve antibiotics without requiring diagnosis-linked data, allowing national and regional comparisons to the WHO-endorsed benchmarks even in countries with resource constraints [10,37]. AWaRe-based antibiotic use measurement provides a population-level signal of antibiotic prescriptions without requiring diagnosis-linked data, allowing national and regional comparisons to the WHO-endorsed benchmark even in countries with resource constraints. The 2025 WHO AWaRe workbook also identifies FDCs of multiple broad-spectrum antibiotics that are not evidence-based and are not recommended for clinical practice, making AWaRe useful for flagging problematic antibiotic products as well as for monitoring use of Access, Watch, and Reserve groups of antibiotics [37].

AWaRe-based measurement is one approach within the broader function of AMC and AMU measurement. Aggregate AMC data can quantify AMC and allow comparison across time, facilities, regions, or countries, while more granular AMU approaches can examine how antimicrobials are prescribed and used in clinical practice. Prescription audits and point-prevalence surveys, for example, can assess prescribing patterns at the patient or facility level and can incorporate clinical information such as indication, antimicrobial selection, route, dose, and duration where these data are available [40]. These approaches are complementary rather than interchangeable: the appropriate measurement method depends on the stewardship question, setting, and availability of reliable data. AWaRe should therefore be interpreted as one component of AMC and AMU measurement rather than as a complete measure of stewardship quality or prescribing appropriateness. Although AWaRe can characterize the distribution of antibiotic use across Access, Watch, and Reserve groups, it does not by itself establish whether an antibiotic was clinically indicated, whether the dose or duration was appropriate, or whether prescribing was concordant with syndrome-specific guidance. Complementary approaches such as prescription audits, point-prevalence surveys, pharmacy dispensing or sales data, and other facility- or syndrome-level measures can provide additional clinical and prescribing context [10,40].

In addition to clinical considerations, antibiotic consumption patterns are shaped by pharmaceutical market incentives and pricing structures, supply-chain reliability, stock-outs of Access antibiotics and prescriber confidence [41]. In India, Watch antibiotics represent a substantial proportion of total sales, and FDC formulations with multiple antibiotics that lack strong scientific justification remain widely marketed [27,36]. These realities illustrate that categorization and measurement alone cannot correct the regulatory, supply-chain, diagnostic, and prescribing conditions that result in antibiotic use inconsistent with stewardship. Regulatory frameworks, pharmaceutical market dynamics, diagnostic capacity, and prescribing incentives often evolve independently rather than in coordination, producing antibiotic use patterns that diverge from stewardship goals. This is consistent with Indian private-sector consumption data showing that Watch antibiotics and non-recommended antibiotic FDCs can remain prominent even where AWaRe classification and regulatory tools exist [29,34,36]. In many LMIC settings, limited electronic prescribing systems and pharmacy-record systems constrain accurate measurement of AMC and AMU, assessment of AWaRe implementation, and comparison with national or WHO-endorsed Access group targets [8,10,33,34].

Operationally, therefore, AMC and AMU measurement should be integrated within the broader AMS governance system. When Access, Watch, and Reserve use is reviewed alongside diagnostic capacity, pharmacy access patterns, stock availability of Access antibiotics, resistance trends, and prescribing workflows, antibiotic use data can identify where stewardship alignment is failing and where targeted intervention is needed [37,40].

### 2.4. Diagnostic Stewardship as the Bridge to Stewardship-Aligned Prescribing

Diagnostic stewardship functions as a critical operational bridge between clinical decision-making and appropriate antimicrobial use within the broader AMS system. Coordinated interventions that improve ordering of appropriate diagnostic tests, quality of clinical specimens, laboratory processing, and interpretation of results directly influence antimicrobial decision-making [1,42,43,44]. As outlined in WHO stewardship guidance, diagnostic stewardship spans the entire pathway, ensuring “the right test for the right patient at the right time to prompt the right action” [44].

In LMIC contexts where limited access to reliable diagnostics often drives empirical broad-spectrum prescribing, strengthening diagnostic infrastructure and workflows can support more targeted therapy. Operationally, this includes prioritizing high-yield, low-cost diagnostics (such as blood cultures and rapid antigen tests where appropriate), improving specimen collection practices, implementing standardized diagnostic algorithms for common syndromes, and ensuring timely reporting of diagnostic test results to clinicians. In LMIC settings, intentional partnerships of prescribers with academic institutions, public health agencies, and clinical laboratories can also support AMS implementation by strengthening AMS training, laboratory quality improvement, routine data collection and review, programme evaluation, and locally relevant implementation research [11,12]. These measures can reduce unnecessary empirical broad-spectrum exposure and improve confidence in narrower-spectrum Access antibiotic prescriptions [42]. When integrated into AMS teams and routine workflows, diagnostic outputs can also function as timely feedback signals, informing prescribing practices, guiding clinical and diagnostic protocol refinement, and strengthening alignment between laboratory capacity and antimicrobial use. In this way, diagnostic stewardship can connect laboratory capacity and diagnostic results with prescribing decisions. Potential outcomes include reduced inappropriate and unnecessary antibiotic initiations, earlier de-escalation to narrower-spectrum antibiotics, and improved alignment with stewardship goals, including appropriate use of Access antibiotics.

### 2.5. AMR Surveillance and Reporting

**Figure 1:**
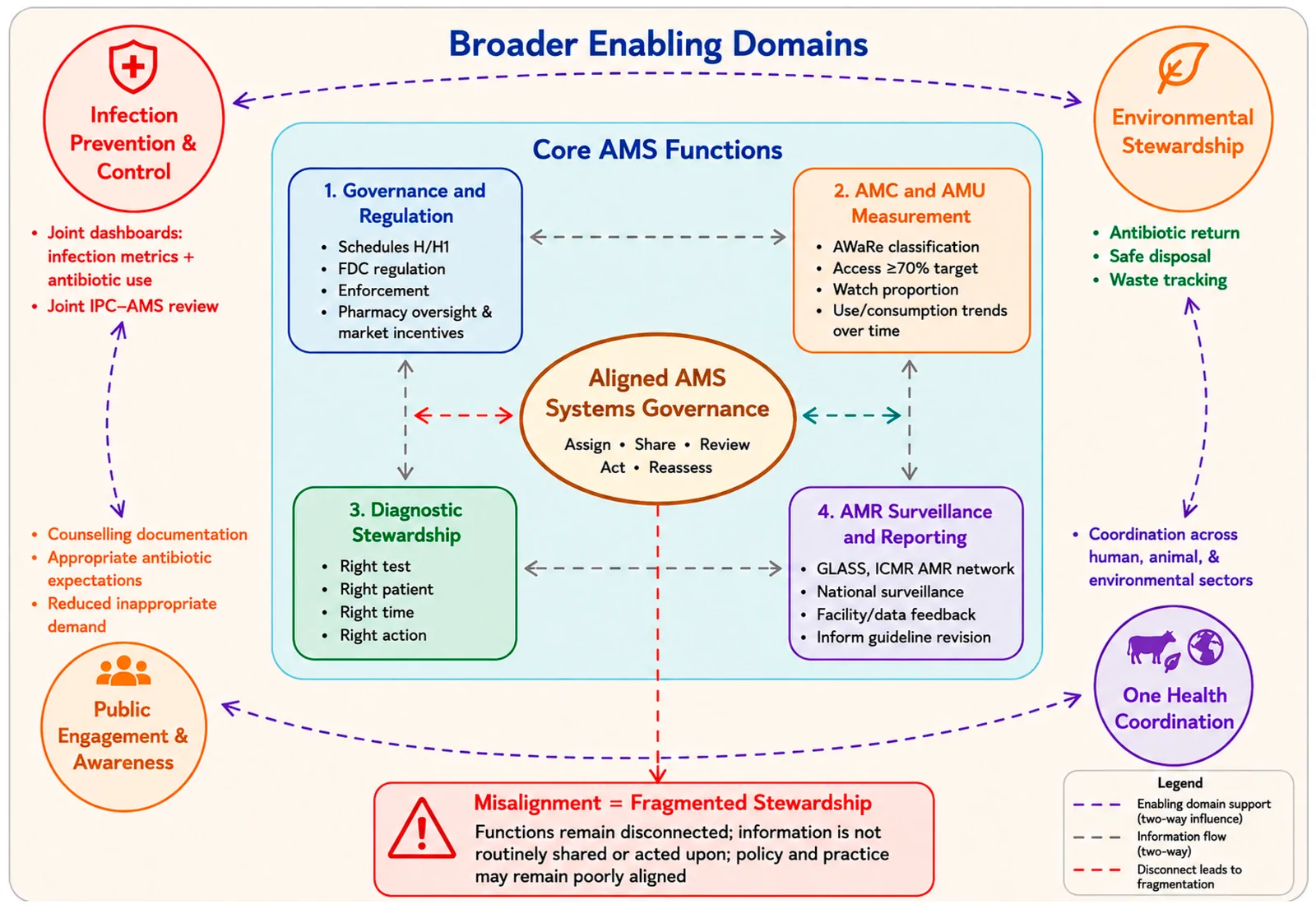
Antimicrobial stewardship as systems governance. Figure 1 presents antimicrobial stewardship (AMS) as a coordinated systems governance framework centered on the Aligned AMS Systems Governance framework. At the center are four interconnected core AMS functions: governance and regulation, antimicrobial consumption (AMC) and antimicrobial use (AMU) measurement, diagnostic stewardship, and antimicrobial resistance (AMR) surveillance and reporting. These functions are linked through continuous feedback loops, reflecting their interdependence in informing antimicrobial use and responses to AMR. Green dashed arrows indicate bidirectional feedback between Aligned AMS Systems Governance and the core AMS functions. Surrounding these core functions are four broader enabling domains: infection prevention and control (IPC), public engagement and awareness, environmental stewardship, and One Health coordination. These domains strengthen stewardship by supporting IPC, informed antimicrobial use, environmental responsibility, and coordination across human, animal, and environmental sectors. Arrows illustrate the continuous interaction between the core AMS functions and the broader enabling domains. When these elements are aligned, responsibilities are defined, relevant information is shared and reviewed, coordinated action follows, and subsequent reassessment provides feedback to the system. When they are misaligned, stewardship may remain fragmented, with functions operating separately rather than as part of a coordinated system.

Taken together, the core AMS functions—governance and regulation, AMC and AMU measurement, diagnostic stewardship, and AMR surveillance and reporting—are interdependent (Figure 1). Surveillance systems close the stewardship feedback loop by generating resistance data that can be compared with patterns of antibiotic use and subsequently used to revise treatment guidelines, diagnostic protocols, and stewardship priorities. The WHO framework, Global Antimicrobial Resistance and Use Surveillance System (GLASS), provides standardized approaches for collecting, analyzing, and reporting national AMR data, including guidance on priority pathogens, reporting structures, denominators, and antimicrobial use metrics [13]. Linking AMC and AMU data with resistance trends enables treatment and policy decisions based on current epidemiological evidence rather than on outdated or incomplete assumptions [13,14]. National surveillance networks, such as the Indian Council of Medical Research (ICMR) AMR Surveillance and Research Network, apply comparable surveillance functions within India by generating laboratory surveillance data that can inform national and institutional treatment guidelines [45,46].

### 2.6. Integrating Core AMS Functions and Broader Enabling Domains

The four core AMS functions operate within four broader enabling domains (Figure 1): IPC, public engagement and awareness, environmental stewardship, including antibiotic waste disposal, and One Health coordination across human, animal, and environmental sectors [3,8,47]. These core functions and enabling domains do not function independently; rather, they operate through continuous feedback loops in which regulatory decisions and market conditions influence antibiotic availability and use; diagnostic findings inform treatment selection; AMC, AMU, and resistance data guide stewardship priorities; IPC reduces preventable infections and corresponding antibiotic demand; public engagement and awareness influence antimicrobial demand and dispensing behavior; environmental stewardship addresses antimicrobial waste and residues; and One Health coordination connects relevant surveillance and policy activity across human, animal, and environmental sectors.

Environmental stewardship is relevant to AMS and must be intentionally integrated with AMS activities because storing unused antibiotics may enable later self-medication, while improper disposal and antimicrobial waste can introduce active residues into wastewater, soil, and other environmental compartments. Environmental contamination may create conditions that contribute to selection and spread of resistance and therefore warrants attention across the antimicrobial lifecycle [3,23,48,49]. In India, the state of Kerala has implemented the ‘nPROUD’ (New Programme for Removal of Unused Drugs) initiative, which facilitates collection of unused or expired medicines from households, pharmacies, and clinics for scientific disposal by high-temperature incineration. nPROUD aims to reduce inappropriate storage and disposal of unused medicines and represents a practical model for incorporating antibiotic-return and safe-disposal processes into AMR mitigation strategies [50]. This initiative aligns with broader One Health stewardship frameworks that emphasize antibiotic disposal and environmental contamination as key stages in the lifecycle of an antimicrobial, misuse and overuse of which increase selection pressure on AMR [3].

The impact of these core AMS functions and broader enabling domains depends on how they are operationalized within routine health system workflows. Stewardship design should specify which person, team, or organization is accountable for each activity; how the activity is incorporated into routine work; what data are collected and shared; and how results are reviewed and acted upon. For example, IPC-linked stewardship can be reviewed through joint dashboards that pair infection metrics with antimicrobial use; public engagement and awareness can be evaluated through documentation of counselling and changes in inappropriate antimicrobial demand or dispensing; and environmental stewardship can be incorporated through protocols for antibiotic return, segregation, and disposal with tracking of waste volumes or pharmacy returns over time [23,49,50]. Without these defined responsibilities, data-sharing processes, and review-to-action mechanisms, stewardship may optimize prescribing on paper while leaving upstream infection transmission, downstream environmental contamination from antibiotic residues, and community antibiotic demand largely unchanged [48]. Weak IPC can increase infection incidence, which in turn leads to greater antibiotic exposure and selection for resistance [10,51]. Poor surveillance can miss or delay detection of resistance trends, weakening guideline credibility because clinicians may lose confidence in recommendations that do not reflect real-world resistance patterns [14,45]. Similarly, inadequate diagnostic capacity drives empiric broad-spectrum prescribing [11,12,42]. Fragmented enforcement of pharmaceutical regulation and prescribing controls may enable inappropriate access to broader-spectrum agents, including Watch antibiotics [8].

Stewardship often fails not because guidance is absent, but because regulatory, diagnostic, prescribing, and surveillance systems (Figure 1) are misaligned, allowing inappropriate antibiotic use to persist despite available guidance [52]. Systems governance coordinates these interdependent AMS functions and domains within health systems by assigning responsibility, defining indicators, and establishing review processes that connect surveillance findings to prescribing guidance, diagnostic protocols, regulatory oversight, IPC priorities, public engagement and awareness strategies, environmental stewardship activities, and where relevant, coordination across One Health sectors. Fidelity to systems governance also complements broader One Health stewardship frameworks that emphasize coordination across sectors and across the antimicrobial lifecycle [3]. Surveillance is therefore not only a reporting function; it is the feedback mechanism that allows stewardship programmes to update practice and refine policy as resistance patterns change at national, regional, and facility levels.

Together, the eight elements illustrated in Figure 1 constitute the proposed Aligned AMS Systems Governance framework. The four core AMS functions are governance and regulation linked to pharmacy oversight and market incentives; AMC and AMU measurement, including AWaRe-based measurement used to assess antimicrobial consumption and prescribing practices, and guide policy updates; diagnostic stewardship linked to appropriate antibiotic selection, including narrower-spectrum, Access-group prescribing; and AMR surveillance and reporting linked to revision of guidelines. The four broader enabling domains are IPC linked to reduced antibiotic demand; public engagement and awareness linked to pharmacy behavior and non-prescription antibiotic demand; environmental stewardship linked to the antimicrobial lifecycle; and One Health coordination, where relevant. When these functions and domains are aligned, responsibilities are defined, relevant data are shared, findings are reviewed across participating functions, and documented changes lead to updated policy and practice. The core-versus-enabling distinction reflects the different roles these elements play within the framework: the core functions directly govern antimicrobial access and use, measure AMC and AMU, inform antimicrobial selection, or detect AMR and use those findings to inform subsequent AMS decisions, whereas the enabling domains shape the conditions in which the core functions operate.

### 2.7. Operationalizing Systems-Based AMS in LMIC Settings

Practical implementation of systems-based AMS in LMIC settings requires phased approaches aligned with local capacity [11]. Initial priorities may include standardizing prescribing criteria for common syndromes in both community and hospital settings, strengthening laboratory quality systems and capacity, improving AMC and AMU measurement, including through AWaRe metrics, and targeting high-volume pharmacies for improved regulatory monitoring [10]. Equally important is integrating data collection into routine work from the start. Early implementation should be led through existing stewardship governance structures, with clear accountability across stewardship, IPC and water, sanitation, and hygiene (WASH), pharmacy, and clinical leadership teams, ensuring that data collection, review, and workflow refinement are embedded into routine operations rather than treated as parallel activities [10,39]. Within the IPC domain, access to water, sanitation, and hygiene services is foundational because inadequate WASH infrastructure limits hand hygiene, environmental cleaning, and other IPC practices, particularly in resource-constrained healthcare settings.

A small set of indicators can be collected reliably, such as syndrome-specific AMS-aligned prescribing rates, proportion of Access versus Watch use, turnaround time for key diagnostic test reporting, blood culture contamination rates, hand-hygiene or bundle compliance, non-prescription dispensing frequency in community pharmacies, and documented patient counselling. These data should be reviewed at regular intervals by multidisciplinary teams and used to refine workflows, target bottlenecks, and assess whether the programme is limiting unnecessary antibiotic exposure and approaching the WHO benchmark of ≥70% antibiotic use being Access antibiotics [9,37,39,51]. More advanced phases may include digital dashboards, real-time prescribing feedback and expanded rapid diagnostic platforms [9,43]. The objective is not replication of high-income country enforcement models, but incremental alignment of the multiple functions and domains of AMS, keeping in mind that effective stewardship depends on systems governance. While this paper focuses on stewardship within human health systems, comprehensive One Health approaches to AMS across human, animal, and environmental sectors and across the antimicrobial lifecycle have been described in detail elsewhere [3].

## 3. Discussion

The central proposition of this review is that AMS effectiveness depends not only on the quality of individual stewardship interventions, but also on whether the surrounding health system can connect those interventions through governance, information, coordinated action, and feedback. Existing guidance provides many of the necessary components: WHO guidance addresses national and facility-level stewardship structures; AWaRe supports standardized classification and monitoring of antibiotic consumption and use; diagnostic stewardship links diagnostic information to treatment decisions; GLASS provides surveillance architecture; and One Health approaches extend stewardship across sectors [3,10,13,37,53]. The contribution of the proposed Aligned AMS Systems Governance framework is to make the operational relationships among these functions more explicit and to treat alignment itself as an implementation objective.

The proposed framework is intended to complement rather than replace existing systems-oriented AMR and AMS frameworks. The WHO Global Action Plan establishes broad strategic objectives across awareness, surveillance, IPC, optimization of antimicrobial use, and investment [2]. The WHO people-centred approach further integrates governance, surveillance, AMC and AMU, diagnostics, AMS, IPC, access, and regulation within a broader health-system response and explicitly recognizes interdependencies among these interventions [7]. WHO policy guidance on integrated AMS activities also emphasizes coordinated national stewardship action under defined governance structures [53]. Published systems-thinking approaches similarly emphasize interdependence, feedback, contextual determinants, and the non-linear effects of stewardship interventions [4,5,6]. The present framework builds on these approaches by focusing more narrowly on how four core AMS functions—governance and regulation, AMC and AMU measurement, diagnostic stewardship, and AMR surveillance and reporting—can be connected operationally within four broader enabling domains.

The distinction between core AMS functions and broader enabling domains is intended as a practical organizing structure rather than an established taxonomy. The four core functions directly govern antimicrobial access and use, measure AMC and AMU, inform antimicrobial selection through diagnostic testing and interpretation, or detect AMR and feed those findings into AMS decisions. The enabling domains influence the context in which those functions operate by affecting infection burden, antimicrobial demand and behavior, environmental antimicrobial exposure, and coordination across human, animal, and environmental sectors. This distinction may therefore help implementers identify which functions directly generate or act on stewardship information and which broader conditions must be addressed for those functions to operate effectively.

Several assumptions underlie the proposed framework. First, AMU is treated as an outcome of interacting clinical, regulatory, market, and social processes rather than solely as the result of individual prescriber behavior. Second, information is assumed to have greater stewardship value when it is reviewed and linked to an identifiable decision or action. Third, the framework assumes that responsibilities can be assigned across organizations and levels of the health system, even when authority is distributed among national, state, facility, pharmacy, laboratory, and professional actors. Finally, the framework assumes that implementation should be adapted to local capacity rather than requiring simultaneous deployment of all eight elements.

These assumptions have practical implications. Implementation may begin with mapping responsible actors, existing data streams, decision points, and bottlenecks in the local antimicrobial-use pathway. A minimum initial system may require only a small set of feasible indicators, provided that those indicators are reviewed at defined intervals and lead to documented action. For example, a district or facility could combine AMC and AMU data, including AWaRe measures where appropriate, with syndrome-specific prescribing audits, diagnostic turnaround times, resistance summaries, and IPC indicators. As capacity increases, these data could be linked through electronic dashboards, pharmacy data, laboratory information systems, or more frequent feedback mechanisms. The objective is not to reproduce resource-intensive stewardship models from high-income settings, but to create an iterative system in which available information is translated into decisions, action, and subsequent reassessment.

Implementation should also recognize that greater alignment can produce trade-offs or unintended effects. Restricting a high-use antibiotic may shift prescribing toward another broad-spectrum agent rather than reduce total inappropriate exposure. Improving access to diagnostics may initially increase testing and documented AMU because previously unrecognized infections are identified. Tightening prescription controls may unintentionally reduce access for patients with legitimate needs if affordable clinical alternatives are not available. Similarly, emphasizing an aggregate target such as the proportion of Access antibiotics could create incentives to substitute within the Access category without improving indication, dose, duration, or patient outcomes. These possibilities reinforce the need to evaluate AMC and AMU, prescribing quality, diagnostic performance, resistance, access, and patient-safety indicators together rather than relying on a single performance measure.

The framework also has implications for equity. Regulation and stewardship interventions designed primarily around formal healthcare facilities may have limited effect where substantial antibiotic access occurs through community pharmacies, informal providers, or private-sector channels. Conversely, aggressive enforcement without attention to affordability, diagnostic access, continuity of care, and reliable supplies of appropriate antibiotics may disproportionately affect patients who already face barriers to care. Systems governance therefore requires attention not only to reducing inappropriate antimicrobial exposure but also to preserving timely access to effective treatment.

Future research should evaluate the proposed Aligned AMS Systems Governance framework as an integrated model rather than examining each element only in isolation. Implementation studies should assess whether deliberate alignment of governance and regulation, AMC and AMU measurement, diagnostic stewardship, and AMR surveillance and reporting—with support from IPC, public engagement and awareness, environmental stewardship, and One Health coordination—improves AMS-aligned antimicrobial use, responsiveness to AMR trends, implementation of stewardship policies, and patient outcomes.

Research should also determine how these elements can be incorporated into routine workflows in hospitals, primary care settings, community pharmacies, and district, regional, or national health systems. Because implementation capacity varies substantially, studies should identify which elements can be introduced using existing personnel and data systems, which require additional laboratory, digital, regulatory, or workforce capacity, and which can be implemented in phases. Evaluation should therefore consider not only effectiveness, but also feasibility, resource requirements, adaptability, and sustainability in different health system contexts [9,10,11].

A practical set of systems governance indicators should also be developed and validated. Potential indicators include whether responsibility for each activity is assigned; whether AMC, AMU, diagnostic, IPC, and resistance data are reviewed together at defined intervals; whether Access, Watch, and Reserve use is monitored over time; whether surveillance findings lead to documented revisions of prescribing guidance, diagnostic protocols, IPC priorities, or regulatory action; and whether agreed changes are subsequently reevaluated. These indicators would help determine whether stewardship elements merely coexist or whether information is shared and used to guide action across participating functions [10,14,37].

Finally, research should determine where coordination across human, animal, and environmental sectors adds measurable value to AMS. Relevant questions include whether linked antimicrobial use and resistance surveillance improves detection or response, how antimicrobial disposal contributes to environmental exposure, and whether findings in one sector lead to policy or practice changes in another. This work would test how the systems governance principles proposed in this review can be applied across the antimicrobial lifecycle while recognizing that implementation priorities and responsible organizations will differ across sectors [3,47].

This review has several limitations. First, it is an analytical narrative review rather than a systematic or scoping review; therefore, the iterative source-identification process has not identified all relevant AMS, AMR, or systems-governance literature. Second, the proposed framework was developed from policy documents, implementation guidance, and selected peer-reviewed literature and has not been prospectively validated as an integrated model. Third, the use of India as the illustrative setting may limit the generalizability of some of the implementation examples discussed. Although several health-system features discussed are relevant to other LMIC settings, the importance of regulatory fragmentation, non-prescription antimicrobial access, diagnostic capacity, pharmaceutical market structure, and surveillance infrastructure will vary across countries and health systems. Fourth, the distinction between four core AMS functions and four broader enabling domains is a proposed practical organization developed for this review rather than an established or validated classification. Finally, the framework does not establish that greater alignment among AMS functions will necessarily reduce AMR or improve patient outcomes; these relationships require prospective evaluation.

## 4. Materials and Methods

This study used an analytical narrative review approach to examine antimicrobial stewardship (AMS) as a systems-governance function rather than solely as a set of interventions directed at individual antimicrobial-prescribing decisions. The review was guided by three questions: (1) which health-system functions directly shape the implementation of AMS; (2) how do these functions interact through the generation, sharing, and use of information to inform AMS decisions and subsequent action; and (3) how might greater coordination and alignment among these functions strengthen AMS implementation, particularly in health systems in low- and middle-income countries (LMICs)? The purpose was not to comprehensively catalogue AMS interventions, but to synthesize policy frameworks, implementation guidance, and peer-reviewed literature relevant to how AMS functions are governed and integrated within health systems.

Sources were identified from major international policy and implementation frameworks and relevant peer-reviewed literature on AMS, antimicrobial resistance (AMR), health-systems governance, and systems thinking. Because the literature was assembled iteratively as the analytical questions and framework developed, rather than through a prospectively defined systematic search, no predefined database search strategy, search terms, publication-date restrictions, or formal study-screening process was used.

Key sources included World Health Organization (WHO) frameworks and guidance addressing the Global Action Plan on AMR, AMS implementation, the Access, Watch, and Reserve (AWaRe) classification, and the Global Antimicrobial Resistance and Use Surveillance System (GLASS). Relevant references within these documents and peer-reviewed publications were subsequently examined to identify additional sources addressing the implementation and interaction of AMS functions. Literature was prioritized when it informed how AMS operates in practice, including regulatory and pharmaceutical governance, antimicrobial consumption (AMC) and antimicrobial use (AMU) measurement, diagnostic stewardship, resistance surveillance, infection prevention and control (IPC), public engagement and awareness, environmental stewardship, and One Health coordination. The WHO people-centred approach to addressing AMR in human health was also reviewed to examine how its approach to strengthening the health-system response to AMR compares with the proposed framework’s emphasis on connecting stewardship functions through defined responsibilities, integration into routine workflows, measurement and information sharing, coordinated review and action, and reassessment of whether those actions produced the intended changes.

Because the objective was to examine how different AMS functions interact within health systems, rather than to evaluate the effectiveness of individual AMS interventions, a formal systematic review and meta-analysis were not undertaken. Sources were considered relevant when they provided evidence, policy guidance, or implementation experience concerning one or more of the AMS functions examined in the review or the relationships among them. Literature focused solely on the clinical efficacy of individual antimicrobial agents, without relevance to AMS implementation or relationships among AMS functions, was outside the scope of the review and was not included.

We examined the selected sources to understand how different AMS functions operate and connect in practice, including who is responsible for each function, what data or information each function generates, and how those data are used to guide antimicrobial prescribing, stewardship activities, regulatory action, or policy decisions. We also examined how one AMS function can inform another; for example, how diagnostic results can guide antimicrobial selection, how AMU data can identify prescribing patterns that warrant AMS action, and how AMR surveillance data can inform treatment guidance and AMS priorities. These connections were used to examine AMS as a coordinated governance process rather than as a collection of separate activities.

Based on an iterative review and analysis of the selected sources, we developed the proposed Aligned AMS Systems Governance framework. The framework distinguishes four core AMS functions—(1) governance and regulation, (2) AMC and AMU measurement, (3) diagnostic stewardship, and (4) AMR surveillance and reporting—from four broader enabling domains: IPC, public engagement and awareness, environmental stewardship, and One Health coordination. We designated the first four as core functions in the proposed framework because they directly govern antimicrobial access and use; measure patterns of AMC and AMU; inform whether antimicrobial treatment is indicated and, when indicated, support appropriate antimicrobial selection through diagnostic testing and interpretation of results; and detect AMR and use those findings to inform subsequent AMS decisions. The other four domains were designated as enabling because they influence the conditions in which the core functions operate. IPC can reduce infection burden and corresponding antimicrobial demand; public engagement and awareness can influence antimicrobial demand and use; environmental stewardship addresses antimicrobial waste and environmental exposure; and One Health coordination connects relevant activities across human, animal, and environmental sectors. The distinction between core functions and broader enabling domains is proposed as a practical way to organize these interconnected functions and domains within a systems-governance framework for AMS, rather than as an established classification of AMS functions and domains.

India was used as an illustrative setting through which to examine the operational relevance of the proposed framework; it was not used as the evidentiary basis from which the framework was derived. India was selected because its health system illustrates several challenges relevant to systems-based AMS, including variation in regulatory implementation, non-prescription antibiotic access, heterogeneous diagnostic capacity, complex pharmaceutical-market dynamics, and expanding but incompletely integrated AMR surveillance. Sources specific to India were identified within the iterative source-identification process described above and were selected when they provided policy or empirical evidence relevant to the AMS functions and implementation conditions examined in the framework. These sources were analyzed for evidence of how the relevant functions were implemented or described in India and for examples of relationships, coordination, or gaps among those functions. Evidence and policy examples from India were therefore used to examine how alignment or misalignment among AMS functions may affect implementation within a complex health system with resource constraints.

We used AI-assisted tools for language editing and clarity during manuscript preparation, and for final modifications to the figure. All content, interpretation, and conclusions were developed, reviewed, and finalized by the authors.

## 5. Conclusions

AMS should be operationalized as a systems governance function rather than confined to individual prescribing interventions. The proposed Aligned AMS Systems Governance framework brings together four core AMS functions—governance and regulation, AMC and AMU measurement, diagnostic stewardship, and AMR surveillance and reporting—with four broader enabling domains: IPC, public engagement and awareness, environmental stewardship, and One Health coordination. Together, these functions and domains reflect the clinical, organizational, regulatory, and cross-sector conditions that shape antimicrobial use and resistance [3,4,5].

The framework’s value lies not merely in whether these elements are present, but in whether they function together. Operational alignment requires defined responsibilities, feasible indicators, routine collection and sharing of relevant data, multidisciplinary review, documented action in response to findings, and subsequent reassessment. Governance and regulation influence antimicrobial access and use; AMC and AMU measurements characterize patterns of antimicrobial consumption and use; diagnostic stewardship connects laboratory capacity with antimicrobial treatment decisions; and AMR surveillance and reporting provide resistance data that can inform treatment guidance and stewardship priorities. IPC, public engagement and awareness, environmental stewardship, and One Health coordination address additional drivers of infection, antibiotic demand, antimicrobial waste, and AMR patterns across human, animal, and environmental sectors.

When these functions remain disconnected, AMS policies and activities may exist without producing coherent implementation or sustained improvement [52]. Reframing AMS as systems governance therefore shifts attention from whether individual AMS activities exist to whether responsibilities are defined, relevant information is shared and reviewed, coordinated action follows, and the effects of those actions are subsequently reassessed. The proposed framework provides a practical structure for examining and strengthening these relationships while complementing existing systems-oriented approaches to AMS and AMR.

## Data Availability

No new data were created or analyzed in this study. Data sharing is not applicable to this article.

## Abbreviations

The following abbreviations are used in this manuscript:

AMS	Antimicrobial Stewardship
AMR	Antimicrobial Resistance
LMIC	Lower- and middle-income countries
IPC	Infection Prevention and Control
GLASS	Global Antimicrobial Resistance and Use Surveillance System
FDC	Fixed-dose combination
AWaRe	Access, Watch, and Reserve
WHO	World Health Organization
ICMR	Indian Council of Medical Research
nPROUD	New Programme for Removal of Unused Drugs
